# Chemical Attributes of Soil Fertilized with Cassava Mill Wastewater and Cultivated with Sunflower

**DOI:** 10.1155/2014/279312

**Published:** 2014-12-23

**Authors:** Mara Suyane Marques Dantas, Mário Monteiro Rolim, Anamaria de Sousa Duarte, Ênio Farias de França de Silva, Elvira Maria Regis Pedrosa, Daniel da Costa Dantas

**Affiliations:** Departamento de Engenharia Agrícola, Universidade Federal Rural de Pernambuco (UFRPE), Rua Dom Manoel de Medeiros, s/n, Dois Irmãos, 52171-900 Recife, PE, Brazil

## Abstract

The use of waste arising from agroindustrial activities, such as cassava wastewater, has been steadily implemented in order to reduce environmental pollution and nutrient utilization. The aim of this study is that the changes in chemical properties of dystrophic red-yellow latosol (oxisol) were evaluated at different sampling times after reuse of cassava wastewater as an alternative to mineral fertilizer in the cultivation of sunflower, hybrid Helio 250. The experiment was conducted at the Experimental Station of the Agricultural Research Company of Pernambuco (IPA), located in Vitória de Santo Antão. The experimental design was randomized blocks with 6 × 5 subplots; six doses of cassava wastewater (0; 8.5; 17.0; 34.0; 68.0; and 136 m^3^ ha^−1^); and five sampling times (21, 42, 63, 84, and 105 days after applying the cassava wastewater), with four replications. Concentrations of available phosphorus and exchangeable potassium, calcium, magnesium and sodium, pH, and electrical conductivity of the soil saturation extract were evaluated. Results indicate that cassava wastewater is an efficient provider of nutrients to the soil and thus to the plants, making it an alternative to mineral fertilizers.

## 1. Introduction

Currently, the world's largest producer of cassava is Nigeria, with 52,403 million tons, followed by Brazil and Indonesia, whose productions were 25,441 and 24,010 million tons, respectively [1]. Of the total cassava production in Brazil, 20% is allocated to the starch industry, which has a high technological level, and about 80% goes to flour mills, which employ traditional methods to obtain table flour [2].

Solid and liquid wastes are by-products generated during the processing of cassava into flour and starch. Compared to other liquid wastes, cassava mill wastewater has high organic load, linamarin, which is a cyanogenic glycoside that can generate hydrocyanic acid (HCN) and minerals [3, 4] and acidic pH [5]; therefore it is considered a highly polluting and toxic waste, presenting a serious threat to the environment and the quality of communities living next to cassava mills.

The indiscriminate disposal of cassava mill wastewater in water bodies can reduce the availability of dissolved oxygen, and its release to the soil can disturb the balance between nutrients, increase salinity, and decrease pH [6]. However, as many nutrients are part of the composition of agroindustrial residues such as cassava mill wastewater, its use may be justified as the reuse of waste for agricultural purposes can promote the economy of natural resources and improve environmental quality [7].

Several studies have been conducted in Brazil in order to demonstrate that cassava mill wastewater can be effectively used as a source of fertilizer, contributing to the development of cultivated plants without any harmful effects on the soil. In a pioneering research with the application of cassava mill wastewater, a significant increase in the levels of exchangeable potassium and phosphorus was observed in soil cultivated with cassava compared to the surrounding control soils, without deleterious effects on the soil and crop [8]. There was an increase in fertility in soil treated with cassava mill wastewater for the cultivation of lettuce and banana [3, 9], with no report of adverse effects on plants.

A study of the possible use of cassava mill wastewater as an agricultural resource under laboratory conditions revealed that the concentration of exchangeable calcium, magnesium, potassium, and sodium increased linearly in three typical soils of Minas Gerais, spodic Orthic Quartzarenic Neosol with moderate A horizon, typical dystrophic yellow Latosol (Xanthic Hapludox) with moderate texture of A horizon, and typical dystrophic red-yellow Latosol (typical Hapludox) very clayey, alic mesoferric, with moderate texture of A horizon, treated with cassava wastewater [10].

In other studies, significant changes were found in the physicochemical properties of soils located in rural areas of Nigeria, caused mainly by the indiscriminate release of untreated cassava wastewater into soils [4, 5]. The authors observed increased acidity and concentration of hydrocyanic acid in these soils. Soil pH is one of the major factors that influence the availability of nutrients and mineralization of organic matter [11].

Thus, in this study, the changes in chemical properties of dystrophic red-yellow latosol (oxisol) were evaluated at different sampling times after the reuse of cassava wastewater as an alternative to mineral fertilizer in the cultivation of sunflower.

## 2. Materials and Methods

**Figure 1:**
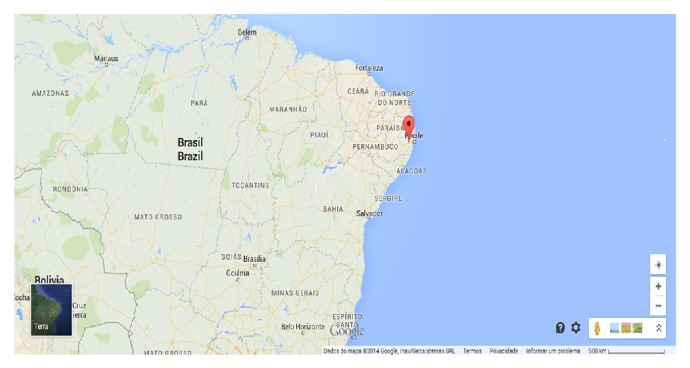
Map of the location of the experimental area at Vitória de Santo Antão, Pernambuco state, Brazil.

The experiment was conducted in Vitória de Santo Antão, Pernambuco, Brazil, located at geographical coordinates 8°6′50′′ south and 35°17′29′′ west and altitude of 146 m above sea level (Figure 1); according to Köppen, the climate fits the type As', called hot and humid tropical with two clearly defined alternating seasons: the rainy season, which occurs from January to June, and dry season, which occurs from July to December.

The soil was classified as dystrophic red-yellow latosol (oxisol) [12], and, in order to characterize its physical and chemical properties, four simple samples were collected from each block using a soil auger at 0.00 m to 0.20 m depth. Subsequently, the samples were air dried, crushed, and sieved through a 2 mm mesh and homogenized to form two composite samples for each block. Physical and chemical properties of the soil to be planted with sunflower (Table 1) were analysed following the methods described in EMBRAPA [13].

A drip irrigation system was used made of flexible 16 mm tubes and emitters spaced at 0.20 m and a flow rate of 1.0 L h^−1^. The irrigations were performed daily, based on crop evapotranspiration (ETc) rate, which was estimated by multiplying the reference evapotranspiration (ETo) by the crop coefficient (Cc) and location coefficient (Lc). Kc values corresponding to each stage of sunflower development were those proposed for sunflower crops by the Food and Agriculture Organization (FAO), and Lc was determined at the beginning of the cycle from the wet area and the coverage area. The ETo was estimated according to the Hargreaves-Samani method using data collected at the Center for Weather Forecasting and Climate Studies weather station (CPTEC/INPE), located in the municipality of Vitória do Santo Antão.

During the entire crop cycle (90 days) an irrigation depth of 357 mm was applied. The preparation of the planting area consisted of plowing and harrowing. A hybrid sunflower Helio 250 was used, and seeding was performed directly by placing 5 seeds per pit and, after their germination, thinning was carried out leaving only one plant per pit. During the experiment, the culturing practices consisted of hand weeding and pest control using natural pesticide (neem extract) and manual scavenging.

The experimental design was a complete randomized block with 6 × 5 subplots; six doses of cassava mill wastewater (0; 8.5; 17.0; 34.0; 68.0; and 136 m^3^ ha^−1^); and five sampling times (21, 42, 63, 84, and 105 days after applying the cassava wastewater), with four replications. Each block contained six experimental plots consisting of four rows 6 m long and spaced 1 m apart, and plants were spaced 0.20 m apart. In each plot, a total of 120 plants were planted, which equates to a planting density of 50,000 plants per hectare. The useful area per plot was equal to 10.4 m^2^; the 52 plants located in the two central rows were evaluated. The two outer rows and two plants from each end of the rows were considered as borders.

The optimal dose of cassava mill wastewater to be applied to the soil was calculated based on the potassium content found in the residue and the soil as well as the requirement of this nutrient by sunflower crops, and according to the recommendation proposed in Ribeiro et al. [14]. The remaining doses were calculated based on the optimal dose multiplied by factors two, four, eight, and sixteen of the initial dose. No mineral fertilization or liming was carried out during the experiment in order to evaluate only the effect of the cassava wastewater on the crop. Doses of cassava wastewater were applied to the soil once, and seeds were planted 15 days later to avoid the toxic effects of cyanide on seed germination.

The cassava mill wastewater that was used in the treatments originated from the pressing of cassava roots used for the manufacture of table flour in a flour mill located in the Municipality of Pombos, PE. Collection, storage, and characterization of physical and chemical parameters (Table 2) were performed according to the methodology proposed by Richards [15].

Characterization of soil chemical properties was performed according to the methodology described in EMBRAPA [13], and the following parameters were determined: levels of exchangeable P, K, and Na were obtained by Mehlich-1 extracting solution (HCl 0.05 mol L^−1^ + H_2_SO_4_ 0.0125 mol L^−1^) followed by colorimetry and flame photometry, respectively; and exchangeable Ca and Mg were obtained by KCl 1 mol L^−1^ extraction solution followed by atomic absorption spectrometry. To determine the pH and the electrical conductivity of the soil saturation extract (CE), saturated paste was prepared according to the method proposed by APHA [16]. CE was determined by electrometric method and the pH of water by potentiometric method [13]. The data of sunflower fresh and dry matter yield of over-ground plant parts was performed according to the methodology described in BENICASA [17]. The obtained data were subjected to analysis of variance and regression with significance levels of 5% for the *F* test.

## 3. Results

Correlation between the factors Doses and Sampling times (*P* < 0.05) was detected for pH and exchangeable Mg and Na (Table 3). All examined chemical soil attributes were correlated (*P* < 0.05) with the isolated factors Doses and Sampling times, except the available phosphorus that was significantly influenced only by the factor Sampling times alone (Table 3).

**Figure 2:**
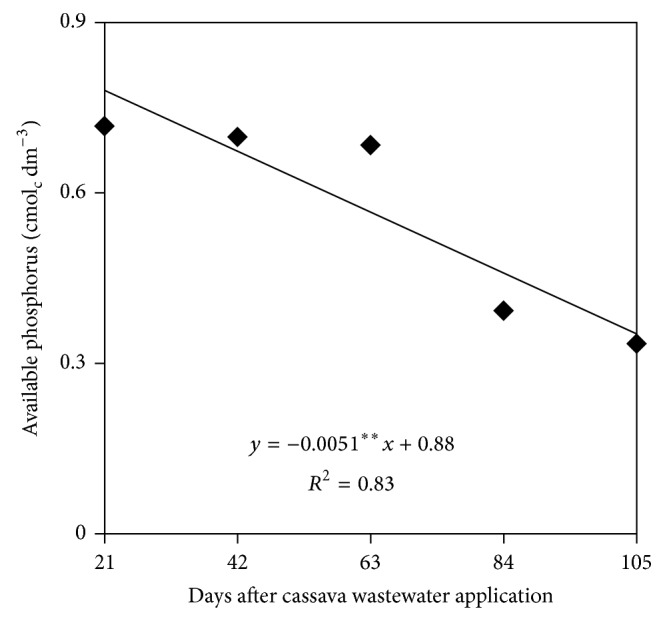
Available phosphorus in soil as a function of sampling time.

The concentration of available phosphorus in the soil (Figure 2) was significantly correlated with the Sampling time, with a decreasing linear trend and values equal to 9.45 mg dm^−3^ and 6.42 mg dm^−3^ at 21 and 105 days after application of cassava wastewater to the soil, respectively, showing a decrease of 32% probably caused by its absorption by plants during the growing cycle.

**Figure 3:**
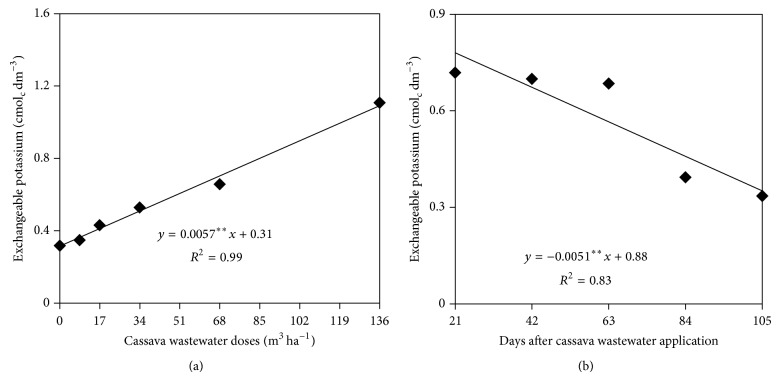
Exchangeable potassium in the soil as a function of cassava wastewater doses (a) and sampling time (b).

The exchangeable potassium in soil was significantly influenced by the isolated factors Doses and Sampling time (Figures 3(a) and 3(b)). The nutrient concentration data, depending on the Doses and Sampling times, followed an increasing and decreasing linear model, respectively. The exchangeable potassium in the soil increased from 0.31 cmol_c_ dm^−3^ to 1.08 cmol_c_ dm^−3^ due to the application of cassava doses that were equal to 0 and 136 m^3^ ha^−1^, respectively; however, we found that this nutrient decreased during the course of sunflower cultivation. At 21 and 105 days after the application of cassava wastewater (DAM), exchangeable potassium concentrations were equal to 0.88 and 0.34 cmol_c_ dm^−3^, respectively.

In addition to exchangeable potassium, the cassava mill wastewater contained considerable concentrations of exchangeable magnesium, sodium, and calcium, in the following order: Mg > Na > Ca.

The exchangeable calcium in the soil was significantly correlated with the factors Doses and Sampling times alone; in the case of the other two exchangeable cations, magnesium and sodium, there was an interaction between the two factors alone.

**Figure 4:**
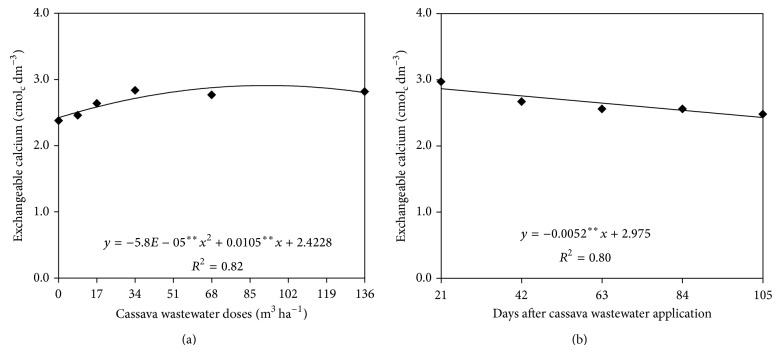
Exchangeable calcium in the soil as a function of cassava wastewater doses (a) and sampling time (b).

In Figure 4(a), one can observe that the data of exchangeable calcium correspond to a positive quadratic polynomial model as a function of the application of increasing doses of cassava to the ground and to a decreasing linear model as a function of the extension of the sampling times (Figure 4(b)). The largest estimated value of exchangeable calcium in the soil, 2.90 cmol_c_ dm^−3^, corresponds to the estimated dose of 90.5 m^3^ ha^−1^, with a decrease in concentration in the soil as higher doses were applied. As for the sampling times (Figure 4(b)), it was found that the concentration of exchangeable calcium in the soil decreased by approximately 22%, from 2.97 to 2.43 cmol_c_ dm^−3^ at 21 and 105 days of cultivation, respectively.

**Figure 5:**
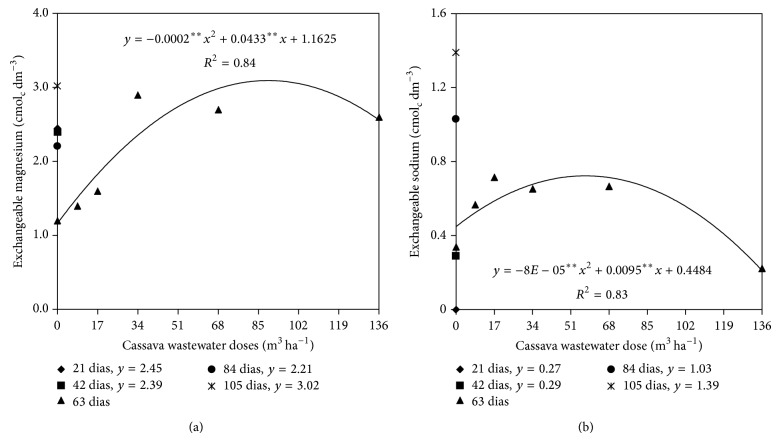
Exchangeable magnesium (a) and exchangeable sodium (b) in soil as a function of cassava wastewater doses and sampling time.

There was a correlation between the isolated factors Doses of cassava water and Sampling times and the levels of exchangeable magnesium and sodium (Figures 5(a) and 5(b)). The data shown in Figures 5(a) and 5(b) indicate that the interaction between the two factors alone was significant only at 63 days after the application of wastewater, with maximum concentrations of exchangeable magnesium and sodium estimated by the quadratic polynomial regression equations, equal to 3.08 and 0.73 cmol_c_ L^−1^, respectively, corresponding to an estimated dose of 108 m^3^ ha^−1^ wastewater and 60 m^3^ ha^−1^ for exchangeable magnesium.

**Figure 6:**
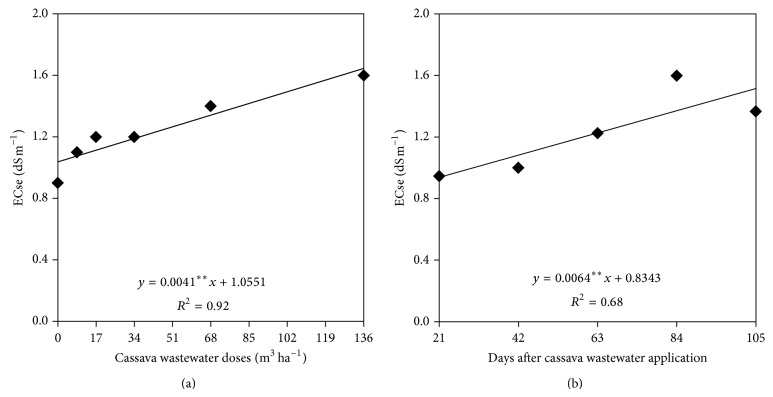
Electrical conductivity of the soil saturation extract as a function of cassava wastewater doses and sampling time.

The electrical conductivity of the soil saturation extract was correlated with the isolated factors doses of cassava wastewater and sampling times, and the data of this attribute fit an increasing linear trend as a function of both doses of cassava wastewater and sampling times (Figure 6(b)). When analyzing Figure 6(a), one can note that the soil CEes ranged from 1.05 to 1.61 dS m^−1^ for doses equal to 0 and 136 m^3^ ha^−1^, respectively, representing a 53% increase.

**Figure 7:**
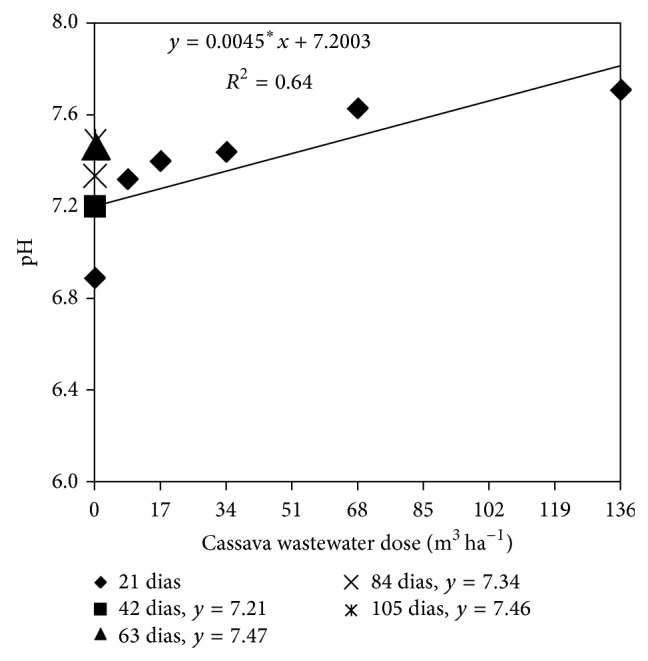
Soil pH as a function of cassava wastewater doses and sampling time.

According to obtained data (Figure 7), a significant correlation between soil pH and increasing doses of cassava wastewater was detected only at 21 days after the application of cassava wastewater (DAM); therefore, the pH data fits a positive quadratic model and depends on the application of increasing doses of wastewater. At 21 days after the application of cassava wastewater (DAM), the soil pH, estimated by the regression equation, in the absence of cassava (0 m^3^ ha^−1^) was 7.20; addition of increasing doses of cassava wastewater increased the pH to a maximum value of 7.81, which corresponds to the application of the maximum dose (136 m^3^ ha^−1^) of wastewater.

**Figure 8:**
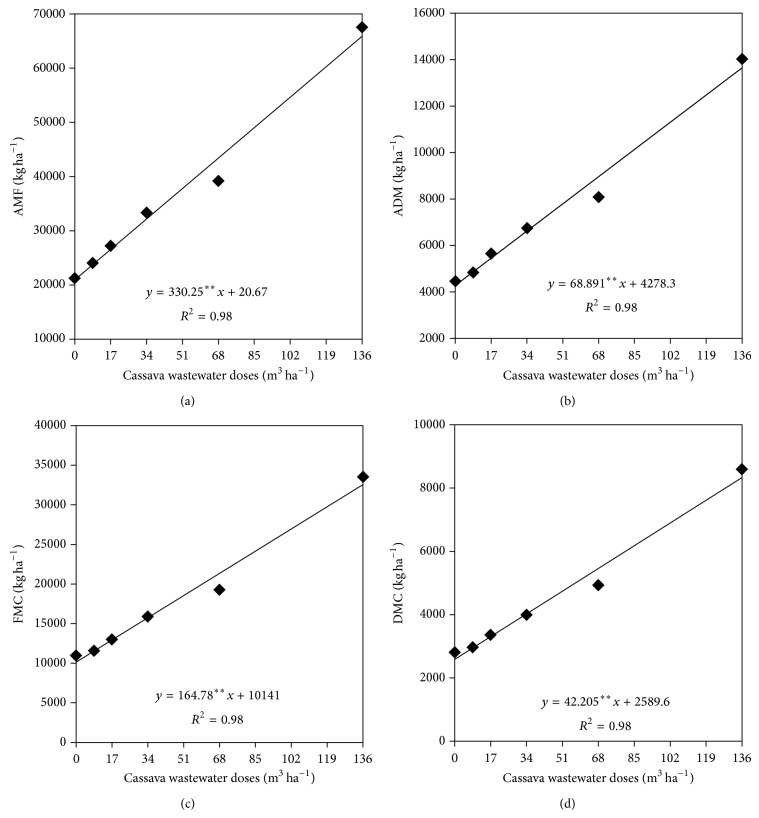
Yield of aerial fresh matter (AFM) (a), aerial dry matter (ADM) (b), fresh matter of capitulum (FMC) (c), and dry matter of capitulum (DMC) (d) in function of cassava wastewater doses.

Data analysis (Table 4) shows that sunflower morphological variables and yield components were significantly (*P* < 0.01) affected by the cassava wastewater doses. The increase in sunflower capitulum and in fresh and dry matter yield of over-ground plant parts showed linear growth as the cassava wastewater increased (Figures 8(a), 8(b), 8(c), and 8(d)). The maximum and minimum profits in fresh over-ground plant parts were predicted by the regression models as 20,967 and 65,881 kg ha^−1^ for 0 and 136 m^3^ ha^−1^, respectively, providing income of 214%.

In relation to dry matter of the over-ground plant parts, the lower yield (4,287 kg ha^−1^) resulted from the absence of cassava wastewater (0 m^3^ ha^−1^), in contrast to the highest dose (136 m^3^ ha^−1^) which provided the highest dry matter of aerial parts representing an increase in 219% (Figure 8(b)). Profits in fresh and dry over-ground plant matters were increased linearly with the lowest and highest yields as response to 0 and 136 m^3^ ha^−1^ of cassava wastewater application, respectively. Under no wastewater application, predicted profits for fresh and dry matter of sunflower capitulum were 10,141 and 2,589 kg ha^−1^, respectively, equivalent to 219.5 and 56.2 g planta^−1^, in contrast to 32.551,1 kg ha^−1^ and 8.328, 9 kg ha^−1^ for fresh and dry capitulum matter fertilized with 136 m^3^ ha^−1^.

## 4. Discussion

Adequate concentrations of available phosphorus in soil range between 21 and 30 mg dm^−3^ [18]. Thus, the content of the available phosphorus in the cultivated soil was below the optimal levels; however, plants showed no symptoms of phosphorus deficiency leading us to believe that the phosphorus from the cassava wastewater supplied the plants with the needed element. It was reported that the contribution of organic phosphorus to plant nutrition is 6% in fertilized soil and increases to 43% in soil treated with mineral fertilizer, which can promote the absorption of this element by plants [19]. In another study, it was observed that the amount of available phosphorus in soil decreases gradually as a function of the number of grown crops and the removal of this nutrient by plants [20]. Besides the uptake by plants, it is argued that a decrease in available phosphorus in soil may be related to the low solubility of phosphorus compounds in soil and the formation of nonlabile compounds in the soil after application of the waste [21].

The levels of exchangeable potassium in the soil above 0.2 cmol_c_ dm^−3^ are considered high or adequate [18], indicating that cassava wastewater used in this experiment served as a source of potassium and supporting the hypothesis that the potassium content in such effluents was sufficient to increase the levels of this cation in the soil and meet the demand of the cultivated sunflower during the experiment. This outcome was expected since cassava wastewater is rich in nutrients (Table 2) especially in potassium, which could explain the increase in exchangeable potassium in the soil due to the application of increasing doses of cassava wastewater. On the other hand, during plant growth throughout the cultivation, as observed for the available phosphorus in soil (Figure 2), the plants extracted available nutrients from the soil solution as needed, thus lowering the concentration of adsorbed potassium in the sorptive soil complex. This decrease can be attributed also to the susceptibility to leaching of potassium ions to the lower soil layers, as mentioned in Duarte et al. [3].

The increase in potassium in the soil due to the application of untreated cassava wastewater to soils in regions of Africa was considerable [4, 5]. Studies conducted in Brazil, aiming at evaluating the use of cassava wastewater as an alternative to mineral fertilizers, revealed that potassium levels in the soil increased to a maximum value of 1.30 cmol_c_ dm^−3^ when 65 m³ ha^−1^ of cassava wastewater was applied to the soil [3].

The exchangeable calcium in the soil showed a similar behavior to the other elements previously discussed with respect to sampling times, indicating that, during the crop cycle, the sunflower plants absorbed calcium provided by the wastewater. Exchangeable calcium levels above 0.4 cmol_c_ dm^−3^ are considered suitable for the cultivation of sunflower [18], showing that the concentration of exchangeable calcium in the soil was high, probably due to fertilization with organic waste completed in previous crops.

Behavior exhibited by the exchangeable sodium and magnesium at 63 days after application of cassava wastewater (DAM) is likely related to a competition between these cations and exchangeable potassium and calcium due to the high concentration of the last two elements in the cassava wastewater (Table 2). When added to soil, they may have been adsorbed by the exchange complex, displacing the exchangeable sodium and magnesium to the soil solution. Once there, the cations, especially sodium, are highly leachable [22]. It is noteworthy that around 60 days after applying cassava wastewater heavy rainfall occurred in the cultivated area. This rain may have promoted the decrease in exchangeable sodium and magnesium, even after applying increasing doses of cassava wastewater, because these cations are highly leachable when present in the soil solution.

In one study, there was an increase in exchangeable calcium in a dystrophic Entisol soil fertilized with different doses of cassava wastewater (0; 12.5; 25.0; 45.0; and 65.0 m^3^ ha^−1^) and planted with lettuce, achieving a maximum concentration of 1.55 cmol_c_ dm^−3^ due to the application of 65 m^3^ ha^−1^ [3]. The levels of exchangeable calcium, magnesium, and sodium in soils located in rural areas close to the Niger Delta, Nigeria, increased during the study period (6 months) due to the disposal of cassava wastewater “in natura” in the environment [5].

Electrical conductivity indirectly represents the total number of cations and anions present in the soil solution [23]. As cassava wastewater is a residue rich in cations and anions, such as K^+^, Ca^2+^, Mg^2+^, Na^+^, PO_4_^−^, and NO_3_^−^, it is believed that the soil application contributed to the increase in electrical conductivity of the soil saturation extract (CEes). An increase in soil electrical conductivity (CEes) in analyzed soils (spodic Orthic Quartzarenic Neosol with moderate A horizon, Xanthic Hapludox with moderate texture of A horizon, and typical Hapludox with very clayey texture of A horizon, alic mesoferric) was due to the use of cassava wastewater, which replaced the mineral fertilization [10].

The increase in electrical conductivity of the soil saturation extract (CEes), caused by the use of solid and liquid waste produced during the processing of cassava as a source of fertilization, was observed when maize was cultivated on latosolic dystrophic Inceptisol located in Minas Gerais, Brazil, and CEes of 0.80 dS m^−1^ was measured when the highest dose of residue was applied [24].

Soil pH is the single factor that affects the most the availability of nutrients to plants. Optimal pH values are between 6.0 and 6.5; in this range, there is a maximum availability of nutrients and organic matter, an increase in the availability of micronutrients, and a reduction of soil acidity, which is a major limitation of agricultural output [18].

Thus, it is likely that the application of cassava wastewater to the soil contributed to an increase in pH by addition of exchangeable cations, especially potassium, magnesium, and calcium, which were present in the wastewater (Table 2). The introduction of bases into the soil favors the increase in pH, as these have the ability to adsorb the sorption complex, shifting elements (hydrogen and aluminum) responsible for the potential acidity of the soil solution; once shifted to the soil solution, aluminum precipitates as Al_2_SO_4_, contributing to the increase in pH [25]. The rise in pH is strongly correlated with the increase in base saturation of the soil [11].

The pH of dystrophic Entisol soil fertilized with 0 and 65 m^3^ ha^−1^ cassava wastewater “in natura” and planted with lettuce rose from 5.08 to 7.72, respectively [25]. However, when studying the effect of the cassava wastewater on chemical, physical, and microbiological characteristics of a typical Hapludox in the coastal plains of the Recôncavo da Bahia, Brazil, the application of cassava wastewater did not alter the pH of the soil [9]. There was an increase in pH of “Oxic Tropudalf” resulting from the application of cassava wastewater to the top soil (0–10 cm), which decreased with increasing soil depth (11–20 cm) and time (5 days). After 10 days of application of the wastewater, the soil pH increased again with increasing soil depth [4].

The increase in soil pH can be caused by the addition of cations present in the waste and a decrease in the reduction potential (Eh), which is related to oxygen consumption during the degradation of organic matter present in the residue; however, the authors emphasize that this increase is transient and reverts to the baseline pH after a certain period of time [26].

The data obtained in this study suggest that cassava wastewater can be used as a source of fertilizer due to its nutritional input, which can minimize the cost of mineral fertilizers and prevent environmental pollution caused by the indiscriminate disposal of this waste.

The increase in fresh and dry over-ground matter of several crops has been related to organic fertilizers use, such as the cassava wastewater. For example, in one study, evaluating the initial growth of maize fertilized with cassava wastewater “raw wastewater” (0, 11.2, 22.4, and 44.8 m^3^ ha^−1^) in two kinds of soil (sand-loam and clay-loam) under greenhouse, significant increases were found in the over-ground fresh matter of plant parts as a result of the increasing cassava wastewater doses application [27]. On the other hand, despite the fact that the maize fresh matter ranged from 15,532 to 17,869 kg ha^−1^ as soil fertilization ranged from 0 to 44.8 m^3^ ha^−1^, the over-ground dry matter of the plant was only affected by the soil kind. In relation to this result, ions in excess in the soil solution, such as potassium, the predominant element in cassava wastewater, could compromise absorption of other essential nutrients for plant growth, such as calcium, magnesium, zinc, and manganese, due to antagonism among them [11].

The sunflower aerial fresh matter production of (65,881 kg ha^−1^) fertilized with the highest cassava wastewater dose (136 m^3^ ha^−1^) was lower than the one found in one research, when growing up four kinds of forages (maize, Sudan sorghum, forage sorghum, and sunflower) in field managed with mineral fertilizers. Among the tested crops, sunflower promoted the highest total fresh matter of the plant parts (83.900 kg ha^−1^), in a sowing density of 60,000 plants ha^−1^ [28]. However, the dry matter of the plant obtained by those authors was lower than the one found in the present research.

The lower the sunflower fresh and dry leaves and stems matter, the higher the panicles fraction [28], which offers higher achene production and, consequently, higher oil yield and nutritional value when the crop is used for silage [29].

In the present work the capitulum dry matter profits when cassava wastewater was used as a substitute for the mineral fertilization were higher than those (3,882.5 kg ha^−1^) obtained in one study when growing the sunflower hybrid BRS 191 in Rhodic Eutrudox (eutroferric Red Latosol) managed with mineral fertilizers [30]. In addition, the responses of capitulum fresh and dry matter presented in our research are similar to those found in another study, when quantifying yield and contribution of achene, leaf, stem, and capitulum in fresh and dry matter of sunflower hybrids Helio 251 and Helio 360 for silage production. They found 1,315.54 and 2,180.89 kg ha^−1^ of capitulum dray matter and 6,473.65 and 11,648.04 kg ha^−1^ of capitulum fresh matter for the hybrids Helio 251 and Helio 360, respectively. These results pointed out that cassava wastewater attended the nutritional exigency of the crop in the text.

## 5. Conclusions

In this study, the changes in chemical properties of dystrophic red-yellow latosol (oxisol) were evaluated at different sampling times after the reuse of cassava wastewater as an alternative to mineral fertilizer in the cultivation of sunflower. Applying cassava wastewater promoted an increase in soil pH, electrical conductivity of the soil saturation extract, and potassium and exchangeable calcium from the soil. The concentration of available phosphorus, exchangeable calcium, and electrical conductivity of the soil saturation extract decreased after applying the wastewater on the soil, while exchangeable magnesium and sodium increased only at 63 days after the application of cassava wastewater (DAM). Thus, cassava wastewater can provide nutrients to the soil and thus to plants, making it an alternative to mineral fertilizers.

## Figures and Tables

**Table 1:** Physical and chemical characteristics of the dystrophic red-yellow latosol (oxisol) before application of cassava wastewater.

Parameters	Values
Sand (g kg^−1^)	597.00
Silt (g kg^−1^)	113.00
Clay (g kg^−1^)	290.00
Texture class	Sandy loam
pH in water	6.60
Organic carbon (g kg^−1^)	28.0
Phosphorus (mg dm^−3^)	7.70
Potassium (cmol_c_ dm^−3^)	0.45
Sodium (cmol_c_ dm^−3^)	0.27
Calcium (cmol_c_ dm^−3^)	2.70
Magnesium (cmol_c_ dm^−3^)	2.80
Aluminium (cmol_c_ dm^−3^)	0.00
Hydrogen + aluminium (cmol_c_ dm^−3^)	2.93

**Table 2:** Chemical and physical characterization of cassava wastewater used in the experiment.

Parameters	Levels
Chemical oxygen demand, COD (mg L^−1^)	66,617
Electrical conductivity (dS m^−1^)	7.27
pH	6.60
Nitrogen (mg L^−1^)	3,064.0
Phosphorus (mg L^−1^)	312.0
Potassium (mg L^−1^)	3,200.0
Calcium (mg L^−1^)	241.9
Magnesium (mg L^−1^)	1,588.2
Sodium (mg L^−1^)	390.0
Sulfate (mg L^−1^)	2,205.0
Chlorides (mg L^−1^)	795.0

**Table 3:** Analysis of variance of the chemical attributes of dystrophic red-yellow latosol (oxisol) fertilized with cassava wastewater after sunflower crop.

Source of variation	Degrees of freedom	Mean square						
		P	K	Ca	Mg	Na	ECse	pH
Block	3	49.60^ns^	0.03^ns^	0.28^ns^	0.67^ns^	0.13^*^	0.47^ns^	0.17^*^
Dose (*D*)	5	22.89^ns^	1.76^**^	0.76^*^	5.98^**^	0.27^**^	0.97^**^	0.16^*^
Sampling (*E*)	4	45.09^**^	0.83^**^	0.87^**^	4.50^**^	4.39^**^	1.57^**^	0.26^**^
*D* × *E*	20	6.39^ns^	0.05^ns^	0.09^ns^	6.94^**^	0.17^**^	0.08^ns^	0.10^**^
CV 1 (%)		53.8	33.65	17.55	21.58	29.81	33.65	3.01
CV 2 (%)		32.8	32.77	13.82	23.32	36.80	31.12	2.63

Overall mean		7.93	0.57	2.63	2.45	0.67	1.24	7.38

^ns^nonsignificant, ^*^significant at 5%, and ^**^significant at 1% by *F* test. P: available phosphorus; K: exchangeable potassium; Ca: exchangeable calcium; Mg: exchangeable magnesium; Na: exchangeable sodium; ECse: electric conductivity of the soil saturation extract.

**Table 4:** Summary of analysis of variance of morphological variables and yield components of sunflower hybrid Helio 250 fertilized with increasing cassava wastewater doses.

SV	DF	Mean square			
		AFM	ADM	FMcap	DMcap
Block	3	70,479,545.48^*^	2,846,290.27^*^	8,489.512^*^	538.10^∗ns^
Dose	5	1,158 × 10^9^^**^	50,404,610.00^**^	115,411.75^**^	7,576.41^**^
CV (%)		13.77	14.77	3.08	9.35

SV: source of variation; DF: degree of freedom; AFM: aerial fresh matter (kg ha^−1^); ADM: aerial dry matter (kg ha^−1^); FMcap: fresh matter of capitulum (kg ha^−1^); DMcap: dry matter of capitulum (kg ha^−1^); ns: not significative; ^*^significative at 5% of probability by *F* test; ^**^significative at 1% of probability by *F* test.

## References

[1] Food and Agriculture Organization of the United Nations (2011). *FAO Production Indices for 2011*.

[2] Araujo J. S. P., Lopes C. A. (2009). Produção de farinha de mandioca na agricultura familiar. *Manual Técnico No.*.

[3] Duarte A. S., Rolim M. M., Silva E. F. F. E. (2013). Alterações dos atributos físicos e químicos de um Neossolo após aplicação de doses de manipueira. *Revista Brasileira de Engenharia Agrícola e Ambiental*.

[4] Osunbitan J. A. (2012). Short term effects of cassava processing waste water on some chemical properties of loamy sand soil in Nigeria. *Journal of Soil Science and Environmental Management*.

[5] Izonfuo W.-A. L., Bariweni P. A., George D. M. C. (2013). Soil contamination from cassava wastewater discharges in a rural community in the Niger Delta, Nigeria. *Journal of Applied Sciences and Environmental Management*.

[6] Wosiacki G., Cereda M. P. (2002). Valorização de resíduos do processamento de mandioca. *Publicatio UEPG-Ciências Exatas e da Terra, Agrárias e Engenharias*.

[7] Laufenberg G., Kunz B., Nystroem M. (2003). Transformation of vegetable waste into value added products: (A) the upgrading concept; (B) practical implementations. *Bioresource Technology*.

[8] Fioretto R. A. (1987). Manipueira na fertirrigação: efeito sobre a germinação e a produção de algodão (*Gossypium hirsutm*, L.) e milho (*Zea mays*, L.). *Semina*.

[9] Da Silva Júnior J. J., Coelho E. F., Sant'ana J. A. D. V., Accioly A. M. D. A. (2012). Physical, chemical and microbiological properties of a dystrophic yellow latosol using manipueira. *Engenharia Agricola*.

[10] Mélo R. F., Ferreira P. A., Ruiz H. A., Matos A. T., Oliveira L. B. (2005). Alterações físicas e químicas em três solos tratados com água residuária de mandioca. *Irriga*.

[11] Fageria V. D. (2001). Nutrient interactions in crop plants. *Journal of Plant Nutrition*.

[12] Empresa Brasileira de Pesquisa Agropecuária (EMBRAPA) (2006). *istema brasileiro de classificação de solos*.

[13] EMBRAPA (Empresa Brasileira de Pesquisa Agropecuária) (1997). *Manual de métodos de análises de solos*.

[14] Ribeiro A. C., Guimarães P. T. G., Alvarez V. H. (1999). *Recomendações para o uso de corretivos e fertilizantes em Minas Gerais*.

[15] Richards L. A. (1954). *Diagnosis and Improvement of Saline and Alkali Soils*.

[16] American Public Health Association (APHA), American Water Works Association (AWWA), Washington Press Club Foundation (WPCF) (1995). *Standard Methods for the Examination of Water and Wastewater*.

[17] Benincasa M. M. P. (2003). *Análise de crescimento de plantas: Noções básicas*.

[18] Malavolta E., Vitti G. C., Oliviera S. A. (1997). *Avaliação do estado nutricional das plantas: Princípios e aplicações*.

[19] Gatiboni L. C., Kaminski J., Rheinheimer D. S., Brunetto G. (2008). Fósforo da biomassa microbiana e atividade de fosfatases ácidas durante a diminuição do fósforo disponível no solo. *Pesquisa Agropecuária Brasileira*.

[20] Gatiboni L. C., dos Santos Rheinheimer D., Claro Flores A. F., Anghinoni I., Kaminski J., de Lima M. A. S. (2005). Phosphorus forms and availability assessed by ^31^P-NMR in successively cropped soil. *Communications in Soil Science and Plant Analysis*.

[21] Rolim Neto F. C., Schaefer C. E. G. R., Costa L. M. (2004). Adsorção de fósforo, superfície específica e atributos mineralógicos em solos desenvolvidos de rochas vulcânicas do Alto Paranaíba (MG). *Revista Brasileira de Ciência do Solo*.

[22] Barros M. F. C., Fontes M. P. F., Alvarez V. H., Ruiz H. A. (2004). Recuperação de solos afetados por sais pela aplicação de gesso de jazida e calcário no Nordeste do Brasil. *Revista Brasileira de Engenharia Agrícola e Ambiental*.

[23] Lee J. (2010). Effect of application methods of organic fertilizer on growth, soil chemical properties and microbial densities in organic bulb onion production. *Scientia Horticulturae*.

[24] Inoue K. R. A., Souza C. F., Matos A. T. (2010). Características do solo submetido a tratamentos com biofertilizantes obtidos na digestão da manipueira. *Tecnologia & Ciência Agropecuária*.

[25] Souza D. M. G., Miranda L. N., Oliveira S. A., Novais R. F., Alvarez V. H., Barros N. F., Fontes R. L. F., Cantarutti R. B., Neves J. C. L. (2007). Acidez do solo e sua correção. *Fertilidade do solo*.

[26] Silva V. S., Garcia C. A., da Silva C. M. (2010). O destino do bagaço da cana-de-açúcar: um estudo a partir das agroindústrias sucroalcooleiras do Paraná. *Revista em Agronegócio e Meio Ambiente*.

[27] Barreto M. T. L., Magalhães A. G., Rolim M. M. (2014). Desenvolvimento e acúmulo de macronutrientes em plantas de milho biofertilizadas com manipueira. *Revista Brasileira de Engenharia Agrícola e Ambiental*.

[28] Oliveira L. B., Pires A. J. V., Viana A. E. S. (2010). Produtividade, composição química e características agronômicas de diferentes forrageiras. *Revista Brasileira de Zootecnia*.

[29] Joner G., Metz P. A. M., Arboitte M. Z. (2011). Aspectos agronômicos e produtivos dos híbridos do girassol (*Helianthus annus* L.) Helio 251 e Helio 360. *Ciência Animal Brasileira*.

[30] Zobiole L. H. S., Castro C., Oliveira F. A. (2010). Curva de crescimento, estado nutricional, teor de óleo e produtividade do girassol híbrido BRS 191 cultivado no estado do Paraná. *Revista Brasileira de Oleaginosas e Fibrosas*.

